# Investigation of *Listeria monocytogenes* in Food in Northwestern Italy (2020–2024)

**DOI:** 10.3390/foods14213788

**Published:** 2025-11-05

**Authors:** Monica Pitti, Matteo Tavecchia, Angelo Romano, Simona Carrella, Giovanna Previto, Daniela Manila Bianchi

**Affiliations:** SC Sicurezza Alimentare, Istituto Zooprofilattico Sperimentale del Piemonte, Liguria e Valle d’Aosta (IZSPLV), Via Bologna 148, 10154 Turin, Italy; monica.pitti@izsplv.it (M.P.); angelo.romano@izsplv.it (A.R.); simona.carrella@izsplv.it (S.C.); giovanna.previto@izsplv.it (G.P.); manila.bianchi@izsplv.it (D.M.B.)

**Keywords:** *Listeria monocytogenes*, virulence factor, NGS

## Abstract

*Listeria monocytogenes* is a foodborne pathogen of significant public health concern due to its high environmental resilience and ability to cause severe infections in vulnerable populations. The objective of the present study is to characterize foodborne strains of *Listeria monocytogenes* isolated between 2020 and 2024 in northwestern Italy. *Lm* was detected through isolation, biochemical confirmation and molecular serogrouping. Next generation sequencing (NGS) analysis was used to characterize the strains in terms of virulence and antibiotic resistance. A total of 39 positive samples were identified from various food matrices, including meat products, fish, cheeses and ready-to-eat foods. The most frequently detected serogroups were IIc and IIa, with a notable presence of the highly virulent IVb group. Next-generation sequencing (NGS) was applied to all isolates, revealing the presence of virulence genes associated with the LIPI-1 island and internalins. In addition to pathogenicity islands, genes related to stress resistance (*clpCEP*, *Gad A*, *GadB*, *GadC*), biofilm production (*agrA*, *flaA*, *degU*, *hfq*) and sortase-mediated anchoring of surface protein (*strA*, *strB*) have been identified. The presence of antibiotic resistance genes was confirmed, with all isolates harboring the *fosX* gene. Moreover, four isolates exhibited resistance determinants against antibiotics belonging to two different classes: tetracyclines (*tetM*) and lincosamides (*lsa*(*A*)). Multilocus sequence typing (MLST) showed that clonal complex CC9 was the most prevalent among the isolates. Further, cgMLST and SNP analyses identified a principal cluster of closely related strains, which were isolated from meat products. These findings highlight the need for continuous surveillance of *L. monocytogenes*.

## 1. Introduction

The genus Listeria currently includes 29 recognized species; only two of these species, *L. monocytogenes* and *L. ivanovii*, are considered pathogens [1]. *Listeria monocytogenes* (*Lm*), a Gram-positive, facultatively anaerobic, non-spore-forming rod, is the causative agent of listeriosis, a serious foodborne infection [2].

*Lm* can be found in a variety of environmental sources, for example, soil, plants, water, silage and animal sources (e.g., cattle, sheep and poultry) [3]. *Lm* is distinguished by its remarkable environmental resilience, which facilitates its growth across a broad temperature spectrum (from 0 °C to 45 °C). Furthermore, it has been demonstrated that it can tolerate pH levels ranging from 4.1 to 9.6 as well as low water activity and high salt concentrations (up to 10% NaCl) [4]. These characteristics enable it to withstand the food preservation processes employed in refrigerated products as well as ready-to-eat (RTE) products [5,6].

One of the mechanisms used by *Lm* to ensure its own survival is the formation of biofilms, which enhance its resistance to environmental stresses and cleaning procedures [7]. Biofilms have been observed to exhibit a high degree of resistance to elevated temperatures, low pH levels, desiccation, UV radiation and salinity [8]. This elevated resistance makes it more difficult to eliminate *Lm*, meaning that biofilms can persist as a source of contamination within the food chain. *Lm* can contaminate food at various stages [8]. It can also persist in equipment that has not been adequately cleaned. Several strategies for eliminating *Lm* from the food chain have been developed and implemented in the food industry, including gamma irradiation, ozone application and phage use [9].

Foods most associated with foodborne listeriosis include RTE products that (i) support the growth of *L. monocytogenes*; (ii) are consumed without receiving any antibacterial treatment (e.g., thermic treatment); and (iii) have a long refrigerated shelf-life [10]. *Lm* has been linked to outbreaks involving various raw and processed foods, including meat products, fish and cheeses [5,11,12,13]. Recent outbreaks were caused by smoked and marinated fish products [14,15,16]. The majority (99%) of the infections caused by *L. monocytogenes* are thought to be foodborne [17].

In the European Union (EU), pursuant to Regulation (EC) No 2073/2005, the presence of *Lm* is not to be detected in 25 g of ready-to-eat foods able to support the growth of Listeria monocytogenes, other than those intended for infants and for special medical purposes, before they have left the control of the producing food business operator where that food business operator is not able to demonstrate that the level of Listeria monocytogenes will not exceed the limit of 100 cfu/g throughout the shelf-life of the product [18].

In the EU, in 2023, listeriosis was the most severe disease, with the highest case fatality and hospitalization rates among reported cases [19]. The case fatality rate of listeriosis (CFR) is approximately 20% to 30% [20]. Between 2019 and 2023, the overall trend of listeriosis cases in the EU showed a marked increase. In 2023, 2952 cases of listeriosis were confirmed in the EU compared to 2778 in 2022 [19]. In Italy, as in the case of the EU, the trend in listeriosis cases appears to be on the rise, although in 2022, a higher number of cases (385) were recorded compared to 2023 (231), mainly due to an outbreak caused by frankfurters [21].

Listeriosis primarily affects immunocompromised individuals, elderly people, pregnant women and neonates, leading to sepsis, meningoencephalitis or fetal loss [22,23,24]. It has been observed that healthy adults may present with self-limiting gastroenteritis [3]. The ability of *L. monocytogenes* to cause disease derives from its invasive power, enabling penetration of diverse mammalian host cells. Following ingestion of contaminated food, *L. monocytogenes* colonizes the gastrointestinal tract and invades the intestinal epithelium [25]. The pathogen subsequently enters the bloodstream via lymphatic and portal routes, reaching the liver and spleen as the primary sites of replication before disseminating more widely. In severe cases, it may breach the blood–brain or placental barriers, leading to central nervous system involvement or fetal infection [26].

*Lm* isolates are classified into four evolutionary lineages (I–IV). Lineages I and II are predominantly implicated in foodborne outbreaks, while III and IV are less prevalent and frequently isolated from ruminants [17]. *Lm* is categorized into 14 serotypes and five major molecular serogroups (IIa, IIb, IIc, IVa and IVb), with serotypes 1/2a, 1/2b, 1/2c and 4b accounting for over 95% of clinical cases [27].

Numerous factors influence the virulence of *Lm*, including ActA (actin assembly-inducing protein), internalins, invasion-associated protein p60, listeriolysin O (LLO), phospholipases and the PrfA virulence regulator [28]. The genes encoding these virulence factors are typically located on four pathogenicity islands (LIPI-1 to LIPI-4) [10,29]. These pathogenicity islands determine the ability to induce listeriosis infection and play a role in bacterial adhesion, internalization, intracellular survival and dissemination. The distributions of LIPI-3 and LIPI-4 were found to be significantly correlated with the high virulence of certain strains of *Lm* [30]. LIPI-3 has been demonstrated to be implicated in the synthesis of listeriolysin S, a pivotal factor in the modulation of the microbial community within the gut lumen [31]. In contrast, LIPI-4 has been shown to be indispensable for central nervous system and placental infection [29].

Since it often causes serious systemic infections, listeriosis often requires antimicrobial therapy. *Lm* exhibits susceptibility to the majority of antibiotics effective against Gram-positive organisms; however, emerging resistance has been increasingly reported [11,32]. *Lm* frequently exhibits resistance to cephalosporins; moreover, resistance to macrolides, fluoroquinolones, fosfomycin and penicillin has also been observed [33]. Recently, there has been an increase in antibiotic resistance among *Lm* isolated from food and environmental sources, particularly antibiotics commonly used to treat listeriosis [34]. Antimicrobial resistance in *L. monocytogenes* has been observed across various food production chains, including beef, pork, chicken, fish and dairy [35]. The antimicrobial resistance of *Lm* may be due to the acquisition of antibiotic resistance genes from other pathogens, particularly *Enterococcus* and *Streptococcus* species [32]. Therefore, it is crucial to monitor the antimicrobial susceptibility of *Lm* in order to track the development of resistance. The presence of MDR pathogens in foods is an increasing public health concern worldwide due to the overuse of antimicrobial drugs in animal feed [36].

In order to characterize *Lm* isolates, several molecular subtyping methods have been developed, including RFLP, PFGE, MLVA, MLST and, more recently, next generation sequencing (NGS) [37]. NGS has been integrated into microbiological analysis for public health surveillance of *Listeria monocytogenes* [38]. The Istituto Zooprofilattico Sperimentale of Piedmont, Liguria and Aosta Valley (IZSPLV) is the official laboratory for the analysis of *Listeria monocytogenes* in foods in northwestern Italy. IZSPLV carries out laboratory analyses on food samples taken during official controls by the local authorities, in accordance with Regulation (EU) 2017/625, to ensure compliance with food safety and public health regulations. In the present study, we analyzed foodborne strains of *Lm* isolated between 2020 and 2024 in northwestern Italy derived from official tests conducted by IZSPLV. This study aimed to characterize the different *Lm* strains to provide high-resolution data on virulence factors, antibiotic resistance and epidemiological links among the isolates.

## 2. Materials and Methods

### 2.1. Sampling

Food samples were obtained as part of the official food safety control activities conducted by local health authorities in northwestern Italy (Piedmont, Liguria and Aosta Valley). A total of 290 samples were analyzed in accordance with the ISO 11290-1:2017 [39].

### 2.2. Isolation

After a primary enrichment, where 25 g of the sample was incubated in 225 mL of Demi-Fraser broth (Biolife, Milan, Italy) at 30 °C for 25 h, a secondary enrichment was conducted by transferring 100 µL of the Demi-Fraser culture into 10 mL of Fraser broth (Biolife, Milan, Italy), followed by incubation at 37 °C for 24 h. A loop of each enrichment broth was streaked onto selective media: Ottaviani Agosti Listeria Agar (ALOA) (Biolife, Milan, Italy) and Polymyxin Acriflavine Lithium Chloride Ceftazidime Aesculin Mannitol Agar (PALCAM) (Biolife, Milan, Italy). After 48 h, typical colonies were subjected to biochemical confirmation using miniaturized galleries (API Listeria, Biomerieux, Marcy-l’Etoile, France).

### 2.3. Serogrouping

A multiplex PCR was utilized for the molecular serogrouping of the isolates (IIa, IIb, IIc, IVa, IVb), targeting the genes *prfA*, *prs*, *lmo0737*, *lmo1118*, *orf2819* and *orf2110* [40,41]. Genomic DNA was extracted using InstaGene Matrix (BioRad, Hercules, CA USA) according to the manufacturer’s instructions. The thermal cycling included a pre-warming step at 95 °C for 15 min, followed by 35 cycles consisting of denaturation at 95 °C for 30 s, annealing at 58 °C for 90 s and extension at 72 °C for 90 s, concluding with a final extension at 72 °C for 10 min. The serogroup assignment was determined based on amplification profiles as detailed in Appendix A Table A1.

### 2.4. Sequencing

NGS analysis was performed according to the protocol of Romano et al. (2023) [42], adapted for *L. monocytogenes*; in particular, during the first phase of DNA extraction, the lysostaphin was not used. DNA extraction was performed using the Extractme Genomic DNA Isolation Kit (Blirt, Gdańsk, Poland) from single colonies grown on Columbia Blood Agar (Biolife, Milan, Italy) for 24 h at 37 °C. Before applying the manufacturer’s protocol, we added a pre-lysis step with an incubation for 30 min at 37 °C with 105 µL of lysozyme (10 mg/mL). DNA quantification was carried out using a Qubit Fluorometer (Thermo Fisher Scientific, Waltham, MA, USA), and library preparation was conducted using the Illumina DNA Library Prep Kit (Illumina, San Diego, CA, USA) according to the manufacturer’s protocol. The sequencing run was performed on an Illumina MiSeq platform (Illumina, San Diego, CA, USA) employing MiSeq V3 chemistry to produce 2 × 151 bp paired-end reads.

### 2.5. Bioinformatics

Data analysis for the detection of virulence factors and antibiotic resistance genes was performed using the CGE tools platform (URL: https://www.genomicepidemiology.org/services/, accessed on 28 July 2025) [43,44,45,46,47,48,49]. A heatmap of the presence or absence of virulence genes was generated using RStudio (version 2024.03.1; RStudio, PBC, Boston, MA, USA) [50]. Phylogenetic relationships were inferred using the SPREAD tool available on the GenPat bioinformatic platform [51] to evaluate cgMLST correlations, while SNP-based comparisons among the strains of the main cluster were performed using CSI Phylogeny 1.4 (available online at https://cge.food.dtu.dk/services/CSIPhylogeny/ accessed on 28 July 2025). A minimum spanning tree (MST) was constructed using Grapetree tools [52]. The distance matrix obtained from the single nucleotide polymorphism (SNP) analysis was constructed using CSI Phylogeny 1.4, including a different matrix strain as the outgroup (Appendix A Table A2), and the related maximum likelihood tree was visualized using ETE 3 [53]. The SNPs workflow, encompassing the processes of SNPs calling, filtering and distance matrix construction, was executed with the following settings: 10x minimum depth at the SNP position, 10% minimum relative depth at the SNP position, 100 bp minimum distance between SNPs (prune), 25 for the minimum SNP quality, 25 for the minimum read mapping quality and 1.96 for the minimum Z-score.

## 3. Results

**Figure 1:**
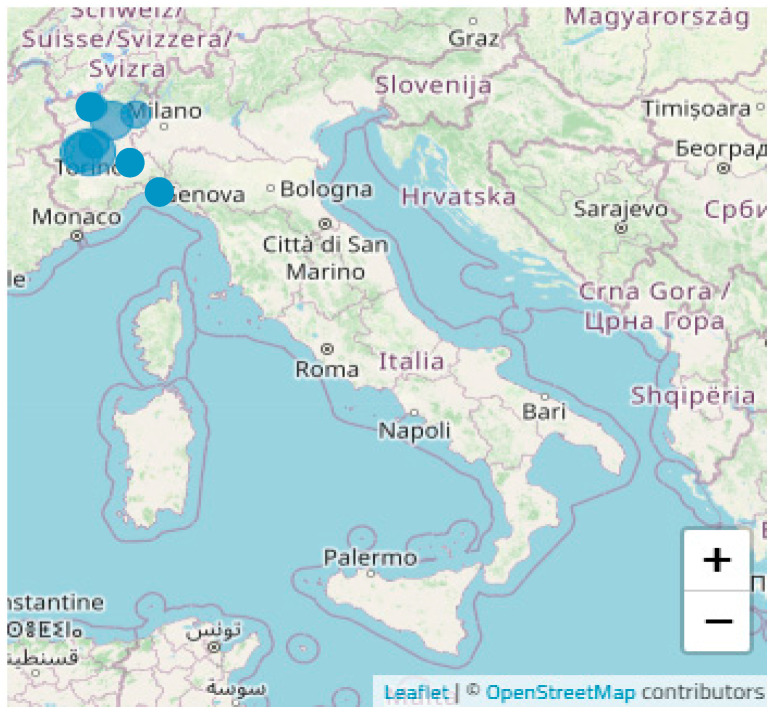
Geographical location of collected samples. The blue dots represent the areas where the samples were collected. This map was created by GenPat with Leaflet-powered OpenStreetMap [54].

Out of 290 food analyses derived from the official control, 39 samples (13.4%) were found to be positive. The samples were collected in the regions of Piedmont, Liguria and Aosta Valley (Figure 1).

**Figure 2:**
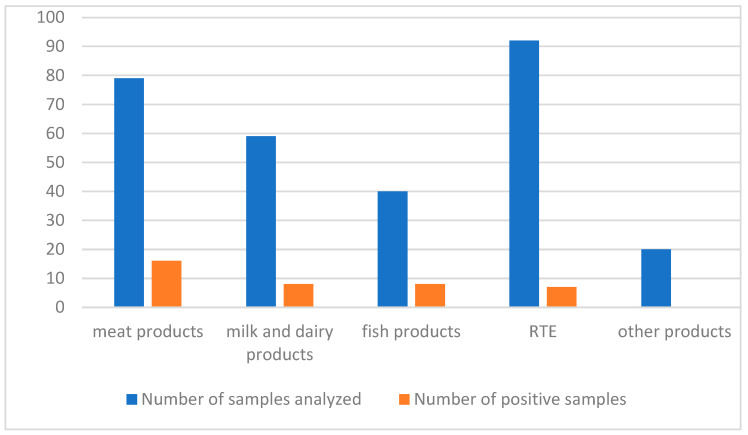
Number of samples analyzed and number of positive samples per food type.

*Lm* was isolated from 16/79 meat products (5.5%), 8/59 cheeses (2.7%), 8/40 fish products (2.7%) and 7/92 ready-to-eat (RTE) foods (2.4%) (Figure 2). Of the 39 positive samples, *Lm* was primarily found in salami (20.5%), sandwiches (10.3%), smoked salmon (7.7%), tome (7.7%) and gorgonzola (2.5%). The serogroups identified were IIa (11 isolates), IIb (7), IIc (12) and IVb (9), with IIc being the most detected.

Resistance genes against fosfomycin (*fosX*) were detected in all isolates (39/39; 100%), while four isolates (4/39; 10.3%) carried tetracycline resistance genes (*tetM*), and only one isolate (1/39; 2.6%) carried a gene conferring resistance to lincosamides (*lsa*(*A*)). Three isolates had resistance genes for both fosfomycin and tetracycline, while one carried *fosX, tetM* and *lsa*(*A*).

**Figure 3:**
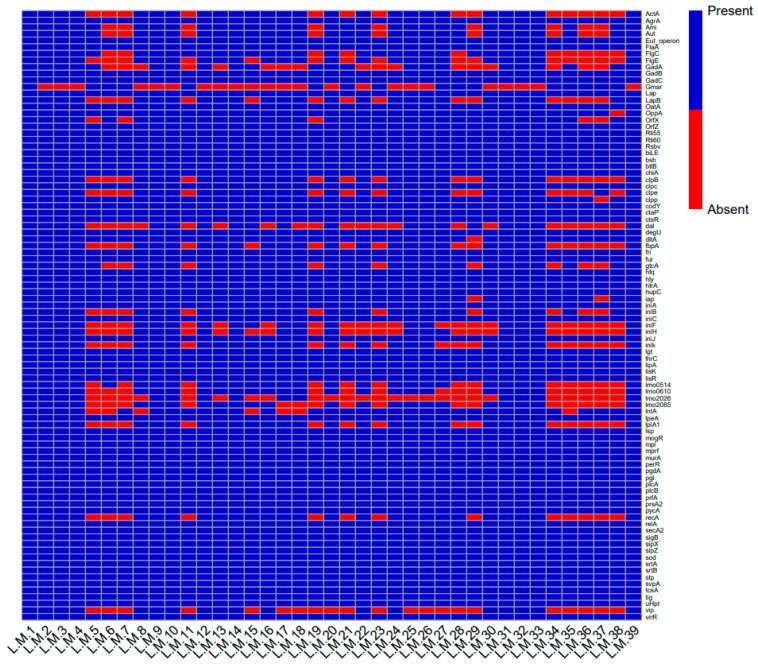
Heatmap of virulence genes in examined isolates. The presence of the gene is represented by blue boxes, while its absence is indicated by red boxes.

The virulence profile revealed that the isolates harbored different LIPI-1 (*hly*, *prfA*, *plcA*, *plcB*, *mpl*, *ActA*) and LIPI-2 (*InlA*, *InlB*, *InlC* and *InlJ*) genes [55]. In addition, other virulence genes have been identified. The genes *agrA*, *flaA*, *degU*, *hfq*, *bilE*, *bsh*, *GadB*, *GadC*, *strA* and *strB* were identified in all strains. Furthermore, the analysis demonstrated that at least one of the *clpc*, *clpe* and *clpp* genes is present in all isolates. In contrast, 12 isolates were found to be deficient in both the *actA*, *vip* and *recA* genes (Figure 3). In this context, only LM1 exhibited all virulence genes; this isolate was identified in 2024 in a sample of fresh meat, and the serogroup identified was IIc (Supplementary Materials Table S1).

Clonal complexes (CCs) were assigned using MLST analysis: CC9 was the most prevalent. The CCs identified were 13 CC9 (33.3%), followed by 4 CC121 (10.3%), 3 CC8 (7.7%) and 3 CC1 (7.7%).

**Figure 4:**
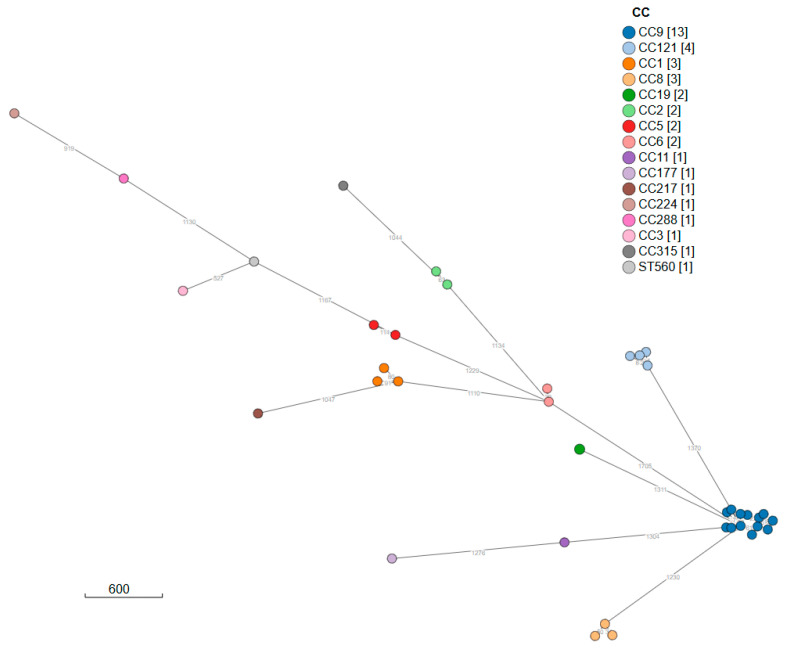
Minimum spanning tree, constructed through cgMLST analysis and visualized with the application SPREAD, allows for the identification of a major cluster A (in blue). Numbers on the branches show the allelic distance between the isolates.

The minimum spanning tree (MS Tree), constructed through cgMLST analysis and visualized with the application SPREAD [56], highlights a major cluster (A) with 13 isolates, all belonging to CC9 (Figure 4).

**Figure 5:**
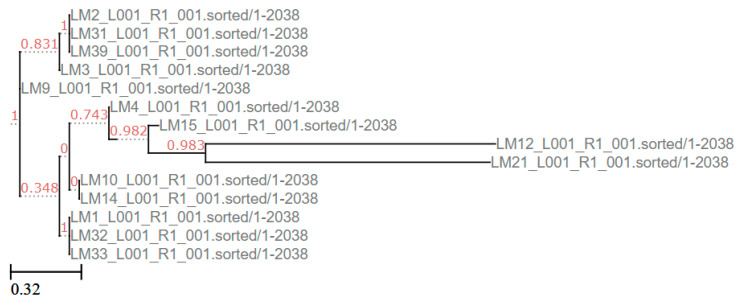
Phylogenetic tree constructed using the distance matrix derived from the SNP analysis, employing the CSI Phylogeny 1.4 and visualized by ETE 3. Support values are shown in red.

For cluster A, a phylogenetic tree was constructed using the distance matrix derived from the SNP analysis. As shown in Figure 5, the strains isolated from cluster A differ by between zero and 1375 SNPs (Appendix A Table A2). Only two samples have a SNP difference > 100 SNPs (LM 15 and LM 12). The LM 21 (isolate not related to cluster A) sequence has been used as an outgroup.

## 4. Discussion

The objective of this study was to characterize *Listeria monocytogenes* isolated over a period of five years in northwestern Italy. The official analysis conducted during this period identified 39 positive samples. *Listeria* contamination was mostly observed in meat preparations (salami), fish products (smoked and raw salmon), milk and dairy products (toma and gorgonzola) and sandwiches [57]. Survival of *Lm* in Italian salami was demonstrated [58]. Notably, the high prevalence of serotype IIc in this study is consistent with that found in other studies conducted in Italy [27,59]. Phylogenetic analysis shows that cluster A contains isolates that are highly correlated. The samples grouped in cluster A, which belong to CC9, were primarily isolated from matrices derived from meat products. Similarly, CC121 is also more closely associated with food [60]. In France, CC9 has been strongly associated with a food origin [61]. In particular, it has been shown that CC9 is directly associated with finished meat products [62].

Serogroup IIa represents the most frequently identified groups in this study. It includes serotype 1/2a, which is frequently reported in food-derived isolates [59]. Similarly, fish products, especially those that are cold-smoked or eaten raw, provided ideal conditions for Listeria to survive and multiply, as shown in previous research [63]. Other foods at risk of contamination by *Lm* include cheeses such as gorgonzola and toma [64,65]. Cheese products provide a favorable environment for the survival and proliferation of *Lm* [66]. Furthermore, RTE sandwiches are particularly at risk, having a complex microbiological composition due to the mixture of ingredients used, which increases the risk of contamination [67].

Strains belonging to serogroup IVb are particularly noteworthy as they are historically linked to multi-country outbreaks of listeriosis [68]. Serogroup IVb predominates among clinical isolates, including those responsible for meningitis [69]. The presence of such highly pathogenic strains underscores the importance of monitoring measures throughout the production and distribution chains. Therefore, NGS has become an important tool for routine surveillance, providing high resolution data on virulence factors, antibiotic resistance genes and genetic relationships among isolates. The results of this study showed that *Lm* isolates carry resistance genes against fosfomycin and, in fewer numbers, against tetracyclines and lincosamides (e.g., clindamycin and lincomycin). Generally, *Lm* is susceptible to almost all antibiotics; nevertheless, resistance has been consistently observed against tetracyclines and fosfomycin [70,71]. Fosfomycin is a broad-spectrum bactericidal antibiotic that blocks peptidoglycan biosynthesis with a putative activity against multidrug resistance bacteria. The fosfomycin resistance gene encodes the FosX protein, which catalyzes the hydration of the antibiotic fosfomycin [72].

Documented case studies have revealed the presence of the gene *tet*(*M*) in *Lm* strains obtained from different food sources [73].

The low frequency of lincosamide resistance observed in this study (2.6%) is consistent with the findings of several strains from other studies describing food processing environments and foods that were found to be non-sensitive to clindamycin [74]. Genes that provide resistance to tetracyclines and lincosamides were identified in one isolate in conjunction with the fosfomycin resistance gene, resulting in a multidrug resistance profile. The resistance genes found in this study confer antibiotic resistance through two different mechanisms [75]: (1) the *tet*(*M*) gene is responsible for encoding a ribosomal protection protein, which, in turn, protects the ribosome from the action of tetracycline, and (2) the *lsa*(*A*) gene encodes efflux proteins that pump one or more of the MLS antibiotics out of the cell.

The detection of a multidrug-resistant isolate in food is particularly concerning, as it suggests that there may be limited therapeutic options for severe listeriosis cases in the future.

After determining the *Lm* antibiotic resistance genes, we set out to identify virulence factors. Most of the virulent genes analyzed exhibited high identity (>90%) to reference genomes, suggesting that the strains examined retain key functions for colonization, invasion and survival in the host. This finding confirms the potential pathogenic capacity of some strains studied. The LIPI-1 (*hly*, *prfA*, *plcA*, *plcB*, *mpl*, *ActA*) genes play a key part in the development of listeriosis, mediating the intracellular cycle of the pathogen [76,77]. In particular, the *hly* (listeriolysin O), *plcA* and *plcB* (phospholipases) and *mpl* (metalloprotease) genes are particularly important for the bacterium’s ability to escape the phagocytic vacuole and spread through lysis from cell to cell [29]. LIPI-1 expression is positively regulated by Prfa, the central transcriptional activator controlling key virulence determinants in LM [78]. A number of other mechanisms, involving environmental, metabolic or stress signals and their processing pathways, contribute to modulate PrfA-dependent expression [79]. The *ActA* gene encodes a surface protein that employs central components of the host cell’s actin cytoskeleton in order to generate actin comet tails for intracellular motility and to facilitate cell-to-cell spread [80]. Then, *ActA* is a key determinant of *Lm* virulence; its absence results in the loss of intracellular motility, rendering the bacterium essentially non-infectious. This study revealed that 30.8% of the isolates were found to lack the *actA* gene and the *vip* gene—another virulence determinant encoding a surface protein that binds to the host receptor Gp96 to promote cellular entry [81]. The simultaneous absence of both genes, in conjunction with the absence of the *recA* gene, which is significant for conferring acid and bile resistance and the ability to adhere to and invade human intestinal epithelial cells [82], suggests that these isolates may possess a reduced capacity to establish infection.

The other pathogenicity island genes identified are on LIPI-2 (*inlA*, *inlB*, *inlC* and *inlJ*), which encode internalins, surface proteins that mediate adhesion and entry into epithelial and other target cells [83]. The occurrence of mutations in the *inlA* gene has been demonstrated to result in a reduction in the bacteria’s capacity to invade host cells [84]. Different internalins have different tissue affinities. For instance, internalin B has been implicated in the process of placental invasion, while internalin A has been observed to induce the internalization of *Lm* into epithelial cells [85]. In particular, InlA has been demonstrated to interact with the host protein E-cadherin, which is located beneath the epithelial tight junctions at the lateral cell-to-cell contacts [86].

In addition to pathogenicity islands, other genes contributing to the pathogenicity of bacteria have been identified. The genes *agrA*, *flaA*, *degU*, *strA*, *bilE*, *bsh*, *strB*, *GadB*, *GadC* and *hfq* were identified in all strains. It has been demonstrated that genes including *GadB* and *GadC* as well as the *GadA* gene that can be found in some isolates play a role in the response to various environmental stresses, including acid tolerance [4]. The response to stress conditions is further facilitated by the *clpc*, *clpp* and *clpe* genes. These genes encode stress proteins that are essential for the regulation of stress survival and intracellular growth [87,88]. The *bilE* and *bsh* genes confer bile resistance, enabling survival in the gastrointestinal tract [89]. In a hostile environment such as the gastrointestinal tract, the role of these genes in facilitating bacterial survival is particularly significant.

The *strA* and *strB* genes encode two sortase enzymes. In *Lm*, *srtA* is essential for the covalent anchoring of surface proteins, including InlA, to the bacterial cell wall [90].

The *agrA*, *flaA*, *degU* and *hfq* genes have been consistently reported in the literature as key regulators of the biofilm formation process [91,92]. The presence of these genes indicates that the isolates studied are able to produce biofilms. This structure represents a persistent source of contamination in food-processing plants, as it protects bacteria from adverse conditions and facilitates their survival in the environment.

## 5. Conclusions

The results obtained provide notable information about *Listeria monocytogenes* isolates detected in various food categories in northwestern Italy during the period from 2020 to 2024. A predominance of serogroup IIc was detected, confirming the association of this serogroup with meat products. Moreover, the detection of serogroup IVb, which is most associated with clinical manifestations, particularly meningitis, represents a risk factor for individuals with a predisposition to infection. The detection of virulence genes involved in the processes of adhesion, invasion, intracellular survival and dissemination in the host highlights the pathogenic potential of these isolates. In addition to detecting the virulence gene, it is important to note the presence of a multidrug-resistant strain. Given the ability *of Listeria monocytogenes* to acquire antibiotic resistance genes from other microorganisms, it is essential to constantly monitor its presence and spread.

Therefore, the use of next generation sequencing is essential for the characterization and surveillance of *Listeria monocytogenes*. NGS analysis provides valuable information on the epidemiology, pathogenic potential and antibiotic resistance profiles of foodborne strains.

Furthermore, from a bioinformatic analytical workflow perspective, we would like to highlight how, due to its greater resolution power, SNP analysis within a cluster derived from cgMLST allows for the identification of strains that may not belong to the same cluster.

The presence of these strains in food products results in a risk for vulnerable consumers and underscores the critical need for rigorous food safety practices at different stages of the food chain.

## Data Availability

The original contributions presented in this study are included in the article; further inquiries can be directed to the corresponding author.

## Supplementary Materials

The following supporting information can be downloaded at: https://www.mdpi.com/article/10.3390/foods14213788/s1, Table S1: list of the characteristics of the 39 positive samples.

## Abbreviations

The following abbreviations are used in this manuscript:

CC	Clonal complex
LIPI	Listeria pathogenicity island
*Lm*	Listeria monocytogenes
MLST	Multilocus sequence typing
cgMLST	Core-genome MLST
MLVA	Multiple-locus variable-number of tandem-repeats analysis
NGS	Next generation sequencing
PCR	Polymerase chain reaction
PFGE	Pulse field gel electrophoresis
RFLP	Restriction fragment length polymorphism

## Appendix A

**Table A1:** Amplification profiles used for serogroup assignment.

Serotype	Target Gene (Expected Bandwidth)						Serogroup
	*lmo 1118* (906 bp)	*lmo 0737* (691 bp)	*ORF 2110* (597 bp)	*ORF 2819* (471 bp)	*Prs* (370 bp)	*prfa* (274 bp)	
1/2a, 3a	−	+	−	−	+	+	IIa
1/2b, 3b, 7	−	−	−	+	+	+	IIb
1/2c, 3c	+	+	−	−	+	+	IIc
4ab, 4b, 4d, 4e	−	−	+	+	+	+	IVb
4a, 4c	−	−	−	−	+	+	IVa
*Listeria* spp.	−	−	−	−	+	−	−

**Table A2:** Distance matrix obtained from the single nucleotide polymorphism (SNP) analysis using CSI Phylogeny 1.4.

	**LM10**	**LM12**	**LM14**	**LM15**	**LM1**	**LM21**	**LM2**	**LM31**	**LM32**	**LM33**	**LM39**	**LM3**	**LM4**	**LM9**
**LM10**	0	1279	2	152	1	1262	2	2	1	1	2	4	2	2
**LM12**	1279	0	1279	1280	1278	1375	1279	1279	1278	1278	1279	1281	1279	1279
**LM14**	2	1279	0	152	1	1262	2	2	1	1	2	4	2	2
**LM15**	152	1280	152	0	151	1259	152	152	151	151	152	154	152	152
**LM1**	1	1278	1	151	0	1261	1	1	0	0	1	3	1	1
**LM21**	1262	1375	1262	1259	1261	0	1262	1262	1261	1261	1262	1264	1262	1262
**LM2**	2	1279	2	152	1	1262	0	0	1	1	0	2	2	2
**LM31**	2	1279	2	152	1	1262	0	0	1	1	0	2	2	2
**LM32**	1	1278	1	151	0	1261	1	1	0	0	1	3	1	1
**LM33**	1	1278	1	151	0	1261	1	1	0	0	1	3	1	1
**LM39**	2	1279	2	152	1	1262	0	0	1	1	0	2	2	2
**LM3**	4	1281	4	154	3	1264	2	2	3	3	2	0	4	4
**LM4**	2	1279	2	152	1	1262	2	2	1	1	2	4	0	2
**LM9**	2	1279	2	152	1	1262	2	2	1	1	2	4	2	0
**min: 0 max: 1375**														
